# Role of Oxidative Stress in the Neural Control of Intra-Renal Hemodynamics in Stroke-Prone Spontaneously Hypertensive Rats

**DOI:** 10.3390/ijms27020558

**Published:** 2026-01-06

**Authors:** Ahmad Ahmeda, Zakarya Ahmeda, Yehia S. Mohamed, Mark G. Rae

**Affiliations:** 1 Department of Basic Medical Sciences, College of Medicine, University of Sharjah, Sharjah 27272, United Arab Emirates; 2 College of Medicine, Newcastle University, Newcastle upon Tyne NE1 7RU, UK; z.ahmeda2@newcastle.ac.uk; 3 Department of Pathological Sciences, College of Medicine, Ajman University, Ajman P.O. Box 34, United Arab Emirates; 4 Department of Microbiology and Immunology, Faculty of Pharmacy (Boys), Al-Azhar University, Cairo 11765, Egypt; 5 Department of Physiology, School of Medicine, Western Gateway Building, University College Cork, T12 XF62 Cork, Ireland; m.rae@ucc.ie

**Keywords:** oxidative stress, renal nerve stimulation, medullary blood perfusion, cortical blood perfusion, hypertension, superoxide dismutase, nitric oxide, catalase

## Abstract

Excessive oxidative stress within the renal medulla is implicated in the development of hypertension, potentially modulated by renal nerve stimulation (RNS). This study examined the effects of RNS on cortical and medullary blood perfusion in Stroke-Prone Spontaneously Hypertensive Rats (SHRSP) under both normal conditions and at varying levels of oxidative stress. Male SHRSP rats were assigned to five experimental groups and subjected to RNS at different frequencies, with infusions of vehicle, tempol, tempol plus catalase (tem + cat), diethyldithiocarbamic acid (DETC), or L-nitro-arginine methyl ester (L-NAME) at the renal cortico-medullary border (CMB). Regional blood perfusion of the renal cortex and medulla (CBP and MBP, respectively) was assessed using Laser-Doppler flowmetry. RNS significantly reduced CBP and MBP by 43 ± 8% and 23 ± 4%, respectively, at 8 Hz. Co-infusion of tempol plus catalase significantly attenuated the RNS-induced reductions in both CBP and MBP. Similarly, DETC infusion mitigated RNS-induced decreases in CBP and MBP. In contrast, tempol alone and L-NAME did not protect against the RNS-induced under-perfusion of the renal cortex and medulla. The results suggest that simultaneous removal of superoxide anion and hydrogen peroxide (H_2_O_2_) can alleviate the reduction in renal blood perfusion caused by RNS, emphasizing a crucial role for H_2_O_2_ in renal hemodynamic regulation. Interestingly, DETC, which is expected to elevate superoxide anion levels, also mitigated RNS-induced under-perfusion, suggesting the presence of a potentially novel indirect protective mechanism that warrants further investigation.

## 1. Introduction

There is clear evidence that activity within renal sympathetic nerves is increased during both the developmental and established phases of hypertension in genetic rat models of hypertension, such as the Spontaneously Hypertensive rat (SHR) and the Spontaneously Hypertensive Stroke-Prone rat (SHRSP) [1], as well as in human essential hypertension [2,3]. Renal-targeted interventions, such as renal denervation, modify blood pressure in selected clinical settings [4]. Thus, basal post-ganglionic sympathetic nerve activity, emotional stress-induced increases in post-ganglionic sympathetic nerve activity and reductions in sodium excretion are all enhanced in SHRs compared with Wistar rats [5,6]. Moreover, renal sympathetic drive increases in human essential hypertension [7]. Additionally, predicted increases in arterial pressure induced by sympathetic stimulation in SHRs were reduced by 30–40% following chronic bilateral renal nerve denervation [8], primarily due to the loss of renal sympathetic drive caused by renal denervation [9].

It has been hypothesized that local redox balance in renal vasculature influences sympatho-vascular coupling, such that elevated reactive oxygen species (ROS) generated by hypertension may weaken or modify the vascular response to sympathetic signals in specific regions. In support of this suggestion, increased production of superoxide and other ROS is strongly associated with hypertension and kidney damage in both animal studies and humans and, further, oxidative stress directly influences neurohumoral systems to affect renal function [10].

Within the kidney, the relative insensitivity of the medullary vasculature to renal sympathetic nerve activity has been well described [11]. Although the mechanism(s) underlying this insensitivity, although of great interest, is/are not well understood, it has been proposed that it may be due, at least in part, to elevated oxidative stress within the renal vasculature [12]. In support of this proposal, the concentration of superoxide anion is elevated in hypertensive rats relative to normotensive rats [13]. Similarly, there is greater macrophage-induced superoxide anion production in hypertensive individuals than in normotensive controls [14].

Mechanistically, superoxide anion influences renal vascular tone by both directly inducing vasoconstriction and by scavenging endothelial nitric oxide (NO), thus reducing NO-mediated vasodilation and affecting local oxygenation and sodium handling in the tubules. These interactions are essential in the renal medulla, where high baseline NO production helps to sustain low vascular tone and maintain medullary blood flow and oxygenation under normal physiological conditions. Experimental reductions in medullary NO (or increases in medullary superoxide anion) decrease medullary blood flow, promote sodium retention, and can elevate arterial pressure, findings that link medullary redox biology directly to blood pressure regulation [15,16].

The SHRSP is a widely used animal model that exhibits exaggerated oxidative stress, with several reports documenting its increased renal superoxide production, NADPH oxidase activity, and oxidative damage relative to normotensive controls. These redox abnormalities occur in concert with altered intrarenal NO signaling and disrupted vascular reactivity, supporting the use of the SHRSP as a suitable model for studying redox–neural interactions in hypertension [17]. 

Despite this conceptual framework, several essential knowledge gaps persist. Firstly, despite general evidence that oxidative stress affects renal vascular function and that increased RSNA is observed in models of hypertension, the specific influence of intrarenal ROS on RNS-induced changes in cortical versus medullary perfusion remains unknown in hypertensive models. Secondly, it is only partially understood how the balance and interaction between NO and ROS determines regional renal neurovascular responsiveness, and whether changes in local ROS levels can uncover or enhance medullary responsiveness to RNS. Finally, prior studies have mainly utilized either systemic interventions or models of chronic disease [18]; thus, fewer studies have combined acute, localized pharmacological ROS manipulation with precise, concurrent measurements of cortical and medullary perfusion during controlled RNS in SHRSP.

We have also previously demonstrated increased NO activity in normotensive and hypertensive rats [19], contributing to reduced renal medullary vascular tone. The SHRSP model used in the aforementioned study represents a model of exaggerated oxidative stress, with elevated superoxide production [20,21].

We therefore propose, based on the current literature, that increased intrarenal oxidative stress in SHRSPs reduces or alters renal medullary vasoreactivity to renal nerve stimulation via interactions with NO bioavailability and ROS-sensitive signaling pathways and that this may lead to region-specific alterations in renal perfusion during sympathetic activation [10]. The current study, therefore, investigated the effect(s) of RNS on renal cortical and medullary blood perfusion in SHRSPs both under normal physiological conditions and following pharmacological manipulation of the degree of oxidative stress within the kidney. Local drug infusion into the kidney was used to scavenge or increase ROS, and the impact of these manipulations on RNS-induced decreases in cortical and medullary blood perfusion was assessed. Our results are discussed with reference to identical experiments conducted in a previous study using normotensive Wistar rats [22].

Study objectives: The present study used localized intrarenal pharmacological manipulation in conjunction with RNS and simultaneous monitoring of cortical and medullary perfusion to test our hypothesis. Specifically, we wanted to:

1. Quantify and compare the immediate effects of RNS on cortical and medullary blood flow in anesthetized SHRSPs under baseline physiological conditions.
2. Determine whether targeted, local scavenging of ROS modifies RNS-induced changes in cortical and medullary blood flow and whether pharmacological strategies that increase ROS levels potentiate or otherwise affect these responses.
3. Compare these results with previously generated results, obtained in normotensive Wistar rats using the same experimental preparation, to identify how oxidative changes associated with hypertension impact neurovascular regulation of cortical and medullary blood flow.

## 2. Results

### 2.1. Effect of Vehicle Infusion on the Cortical and Medullary Blood Perfusion (CBP & MBP, Respectively)

Table 1 presents the baseline levels of average blood pressure, heart rate, and cortical and medullary blood perfusion in SHRSPs. Vehicle infusion into the cortico-medullary border did not significantly alter these measures.

**Figure 1:**
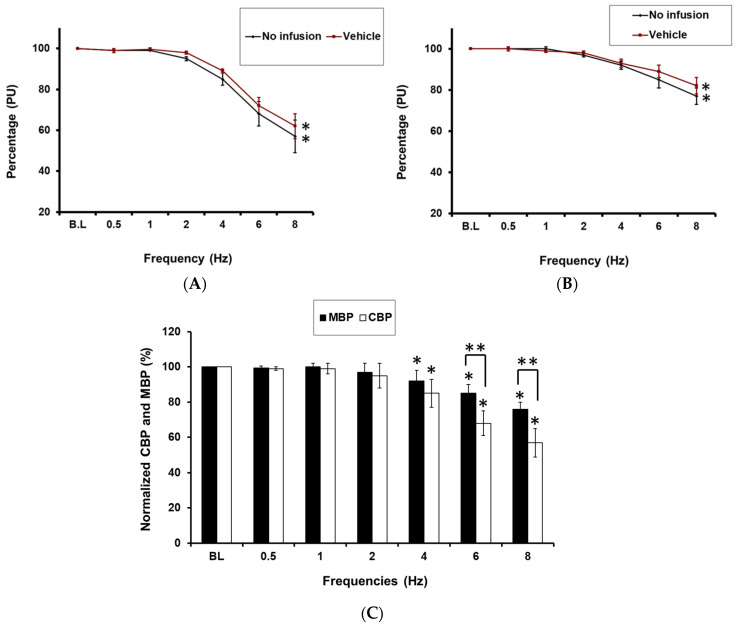
(**A**): Effect of vehicle infusion on cortical blood perfusion in response to RNS. * = *p* < 0.05, compared with the baseline. (**B**): Effect of RNS on medullary blood perfusion before and after vehicle infusion * = *p* < 0.05, compared with baseline. (**C**): Comparison between cortical and medullary blood perfusion in response to RNS. RNS affected the cortical more than the medullary blood perfusion at all frequencies ≥4 Hz (* indicates *p* < 0.05 relative to baseline values, and ** indicates *p* < 0.05 of CBP relative to MBP).

RNS induced a frequency-dependent reduction in both CBP (by 43 ± 8% at 8 Hz; *p* < 0.05; *n* = 10; Figure 1A) and MBP (by 23 ± 4% at 8 Hz; *p* < 0.05; *n* = 10; Figure 1B) from baseline. These parameters were unaffected by vehicle infusion into the cortico-medullary border. Notably, there were significant differences in how RNS impacted cortical and medullary blood perfusion in the SHRSP (Figure 1C).

### 2.2. Effect of Renal Infusion of Tempol on CBP and MBP

Baseline levels of average blood pressure, heart rate, and cortical and medullary blood perfusion for this group are presented in Table 1. Baseline readings for all hemodynamic factors measured throughout these experiments remained relatively stable.

**Figure 2:**
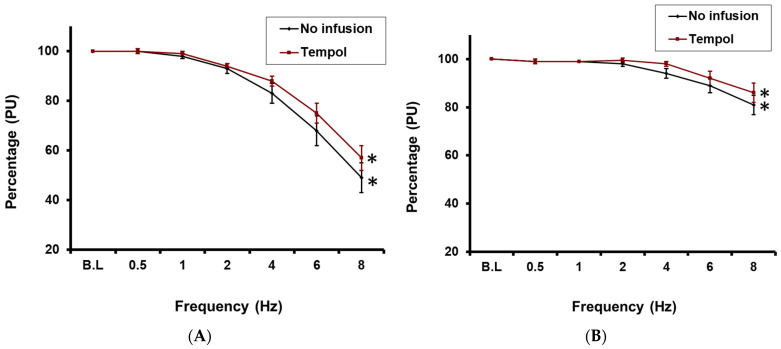
(**A**): Effect of RNS on cortical blood perfusion before and after tempol infusion * = *p* < 0.05 compared to baseline. (**B**): Effect of RNS on medullary blood perfusion before and after Tempol infusion * = *p* < 0.05 compared to baseline.

Before the infusion of tempol, RNS significantly reduced CBP by 51 ± 7% at 8 Hz from baseline (*p* < 0.05; *n* = 10; Figure 2A). When tempol was infused into the cortico-medullary border, RNS still evoked a significant reduction in CBP (43 ± 5% at 8 Hz from baseline; *p* < 0.05; *n* = 10; Figure 2A), but the magnitude of the CBP reduction did not differ significantly from that evoked under control conditions.

Under control conditions, RNS also significantly decreased medullary blood perfusion at frequencies of 6 Hz and above, with a maximum reduction of 19 ± 4% at 8 Hz relative to baseline (*p* < 0.05; *n* = 10; Figure 2B). However, similar to CBP, in the presence of tempol, RNS did not significantly attenuate the RNS-mediated decrease in MBP responses relative to control (maximum decrease of 14 ± 4% at 8 Hz; *p* < 0.05 compared to baseline; *n* = 10; Figure 2B).

### 2.3. Effect of Renal Infusion of Tempol and Catalase on CBP & MBP

Baseline levels of average blood pressure, heart rate, and cortical and medullary blood perfusion for this group are presented in Table 1. Baseline readings for all hemodynamic factors measured throughout these experiments remained relatively stable.

**Figure 3:**
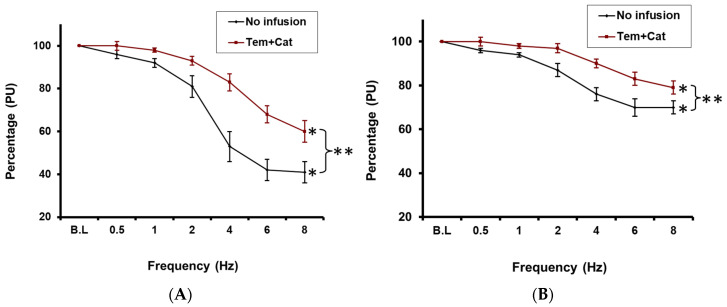
(**A**): Effect of the RNS on cortical blood perfusion before and after tempol plus catalase infusion (Tem + Cat) * = *p* < 0.05 compared to baseline. (**B**): Effect of RNS on the medullary blood perfusion before and after intra-medullary infusion of tempol plus catalase (Tem + Cat) * = *p* < 0.05 compared to baseline. ** indicates *p* < 0.05 when comparing responses during Tem + Cat infusion vs. control).

RNS evoked a significant frequency-dependent reduction in CBP under control conditions, reaching a maximum 59 ± 6% decrease relative to baseline at 8 Hz (*p* < 0.05; *n* = 10; Figure 3A). In the presence of both tempol and catalase however, although RNS-evoked CBP fell significantly relative to baseline values (maximal reduction 40 ± 5% at 8 Hz; *p* < 0.05; *n* = 10; Figure 3A), the response to RNS was significantly attenuated across all frequencies, with roughly a 20% reduction in the maximum cortical blood perfusion decrease, compared to control values (*p* < 0.01; Figure 3A).

Under control conditions, RNS also evoked a significant frequency-dependent decline in MBP, peaking at 30 ± 5% at 8 Hz relative to baseline (*p* < 0.05; *n* = 10; Figure 3B). In the presence of both tempol and catalase however, although RNS still induced a significant frequency-dependent decrease in MBP relative to baseline (*p* < 0.05; *n* = 10; Figure 3B), responses were significantly attenuated compared to control values at all frequencies examined, with a maximum decrease of only 21 ± 5% at 8 Hz, compared to baseline (*p* < 0.01; Figure 3B).

### 2.4. Effect of Renal Infusion of DETC on Cortical and Medullary Blood Perfusion (CBP & MBP, Respectively)

Table 1 presents baseline values for all measured hemodynamic factors, which remained stable throughout the experiments.

**Figure 4:**
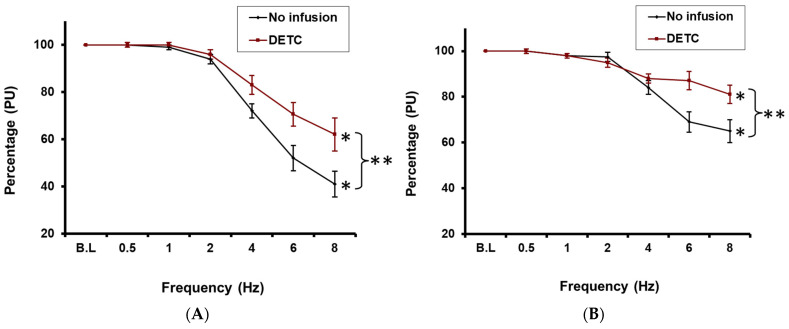
(**A**): Effect of RNS on cortical blood perfusion before and after DETC infusion * = *p* < 0.05 compared to baseline. (**B**): Effect of RNS on medullary blood perfusion before and after DETC infusion * = *p* < 0.05 compared to baseline. ** indicates *p* < 0.05 when comparing responses during DETC infusion vs. control).

In these experiments, under control conditions, RNS again evoked a significant frequency-dependent reduction in CBP, with a maximum decrease of 59 ± 9% at 8 Hz relative to control (*p* < 0.05; *n* = 10; Figure 4A).

Although following a 90 min infusion of the SOD inhibitor, DETC, into the cortico-medullary border, RNS again evoked a similar significant frequency-dependent decline in CBP (38 ± 12% at 8 Hz relative to baseline; *p* < 0.05; *n* = 10; Figure 4A), this maximum decrease was significantly less (~20%) than that observed in the absence of DETC (Figure 4A).

Medullary blood perfusion decreased by 35 ± 5% at 8 Hz compared to baseline values in response to RNS under control conditions (*n* = 10; *p* < 0.05; Figure 4B). However, although MBP was still significantly reduced following RNS in the presence of DETC relative to baseline (19 ± 8% at 8 Hz; *p* < 0.05; *n* = 10; Figure 4B), the RNS-induced reduction in MBP was significantly attenuated at 6 Hz and above, compared to the reduction seen without DETC at 6 Hz and above, only reaching a maximum of 19 ± 8% at 8 Hz compared to 35 ± 5% at 8 Hz in the absence of DETC (*p* < 0.01).

### 2.5. Effect of Renal Infusion of L-NAME on CBP & MBP

Table 1 presents baseline levels of average blood pressure, heart rate, and cortical and medullary blood perfusion. Baseline readings for all hemodynamic factors measured throughout these experiments remained relatively stable.

**Figure 5:**
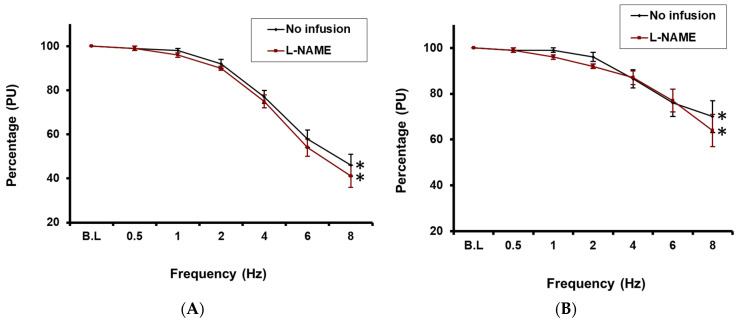
(**A**): Effect of the RNS on cortical blood perfusion before and after L-NAME infusion * = *p* < 0.05 compared to baseline. (**B**): Effect of RNS on the medullary blood perfusion before and after L-NAME infusion * = *p* < 0.05 compared to baseline.

Under control conditions, RNS induced a significant frequency-dependent reduc-tion in CBP, with a maximal decrease of 53 ± 9% below baseline values at 8 Hz (*p* < 0.05; *n* = 10; Figure 5A). In the presence of L-NAME, RNS again evoked a significant reduction in CBP (59 ± 9% at 8 Hz relative to baseline; *p* < 0.05; *n* = 10; Figure 5A). Notably however, this reduction did not differ significantly from the reduction evoked by RNS under control conditions.

RNS also produced a significant frequency-dependent reduction in MBP, which peaked at 30 ± 7% at 8 Hz relative to baseline (*p* < 0.05; *n* = 10; Figure 5B). However, as with the CBP data, following the infusion of L-NAME, subsequent RNS elicited similar reductions in MBP to those observed in the absence of L-NAME, with a maximal medullary blood perfusion decrease of 36 ± 7% at 8 Hz relative to baseline (*p* < 0.05; *n* = 10; see Figure 5B).

## 3. Discussion

This study examined the effect of RNS on renal hemodynamics, focusing on blood perfusion in the cortical and medullary regions of SHRSPs. We also investigated the roles of ROS and NO in regulating renal blood perfusion during RNS.

Initial control experiments assessed the effect of vehicle administration into the CMB, confirming the consistency of our setup and the inability of the vehicle to influence baseline or RNS-mediated changes in blood perfusion in the region.

These control experiments enabled us to compare, for the first time, the impact of RNS on regional renal blood perfusion in SHRSPs. Results showed that RNS had a weaker vasoconstrictor effect on cortical and medullary blood vessels of hypertensive rats compared to normotensive rats [22]. This suggests that factors in the hypertensive kidney might affect its response to RNS by altering vasculature sensitivity to constriction.

Superoxide free radicals, which are elevated in the renal medulla of hypertensive rats [12,23], may influence renal vasomotor tone. These free radicals significantly constrict vascular smooth muscle [24,25,26,27] and decrease NO availability [28,29,30]. Both mechanisms, separately or together, may reduce cortical and medullary blood perfusion responses to RNS in SHRSPs because their renal blood vessels are more contracted than those in normotensive controls. In SHRSPs, resting blood pressure is higher, and heart rate is lower, than in normotensive rats, contrary to previous findings [22].

This study confirmed that RNS induces less vasoconstriction in pre-constricted vessels than in normotensive rats.

The medullary vasculature appears to be less sensitive to RNS than cortical vasculature, aligning with our previous findings in normotensive rats. This reduced sensitivity may be due to higher NO concentrations in the kidney’s deeper regions, protecting it against under-perfusion from RNS. Zhou & Cowley (1997) indirectly supported this hypothesis with their finding that NO concentration in the renal medulla was significantly greater than in the renal cortex [31].

### 3.1. Tempol Study

We investigated how tempol, a potential SOD scavenger, affects the vasoconstricting effects of superoxide anions on CBP and MBP in response to RNS. Previous reports indicated that acute intravenous treatment with tempol decreased BP and HR in SHR [32]. Local application of tempol into the cortico-medullary border enhanced medullary perfusion, suggesting that it reduces vascular tone by eliminating superoxide anions [33].

Local administration of tempol at the cortico-medullary border in SHRSPs evoked a greater rise in basal MBP than in normotensive rats [22,34]. This difference may be due to the elevated concentration of superoxide anions in hypertensive animals, which likely inactivate any released NO [35].

In the presence of tempol, the significant RNS-mediated reductions in either CBP or MBP were not attenuated compared to controls. These findings imply that the observed insensitivity of the medulla and cortex in hypertensive rats to RNS is unlikely to be due to an elevated superoxide anion concentration in the kidney.

Tempol significantly reduced the vasoconstrictor effect of RNS on CBP at 6 and 8 Hz in normotensive rats. This suggests that the cortex, but not the medulla, is insensitive to tempol in hypertensive rats compared with normotensive rats.

One explanation for the insensitivity of renal perfusion to tempol, particularly in the medulla, is that basal NO release may be maximal in this tissue. If so, reducing superoxide with tempol, which prolongs the half-life of NO, might not effectively counter the effects of RNS on MBP and CBP. However, our findings using L-NAME (discussed later) suggest that this is unlikely. Another possibility is that tempol inhibits renal sympathetic nerve activity, as indicated in previous studies [32,36]. This inhibition may reduce the release of noradrenaline from the renal nerves.

### 3.2. Tempol Plus Catalase Study

Another ROS produced in elevated quantities within blood vessels of hypertensive animals is H_2_O_2_. This compound is a breakdown product of the superoxide anion [37,38], and its production can also be stimulated by tempol. H_2_O_2_ may induce renal vasoconstriction [23], potentially counteracting the vasodilatory effects of NO, thereby mimicking the influence of superoxide anions on the vasculature.

A previous study co-infused tempol and catalase to inhibit the main vasoconstrictors, superoxide anions, and H_2_O_2_. The results showed that RNS-induced decreases in MBP and CBP in normotensive rats were significantly smaller than those evoked by tempol alone [22].

The current study also examined the effects of co-infusing tempol and catalase, this time into the renal medullae of hypertensive rats. Although our findings mirrored those observed in normotensive rats, the degree of attenuation was significantly greater than that achieved with tempol alone in both the cortex and medulla. This suggests that the additional infusion of catalase effectively inhibited the substantial vasoconstrictor activity induced by RNS, likely mediated by H_2_O_2_.

The combined use of tempol and catalase demonstrated a markedly greater protective effect against the vasoconstrictor actions of RNS than administration of tempol alone. These results provide further, albeit indirect, evidence for the role of NO in safeguarding the renal medulla against the detrimental effects of RNS-induced under-perfusion.

### 3.3. DETC Study

To further investigate medullary insensitivity to RNS in SHRSPs, we examined the effect of increased oxidative stress by locally administering the SOD inhibitor, DETC. We hypothesized that this would raise superoxide anion levels enough in the renal medulla to inhibit any vasodilatory effects of NO. We anticipated that this would negate the increases in renal medullary or cortical perfusion post-RNS relative to control values in the absence of the inhibitor.

We found that DETC infusion improved, rather than intensified, the effects of RNS on medullary and cortical blood perfusion. Notably, there was a significant impact on medullary perfusion with RNS at 6 and 8 Hz. This finding was unexpected, as one would have anticipated that DETC would further decrease both medullary and cortical blood perfusion in response to RNS, given its proposed mechanism of action. Our results contrast with previously reported findings [39], which demonstrated that DETC significantly increased renal sympathetic nerve activity following local application of DETC to renal sympathetic nerves in the area between the recording electrodes and the ganglion.

One possible, albeit unlikely, explanation for our observations is that DETC infusion may have maximally constricted the renal vasculature. This may explain the decrease in MBP that we observed in our previous study [34], suggesting that RNS could not further reduce perfusion. Alternatively, DETC may be acting non-specifically to prevent under-perfusion of the renal cortex and medulla that can result from RNS. In a prior study, the effects of RNS on normotensive animals in the presence of DETC were consistent with these current findings [22].

### 3.4. L-NAME Study

The present studies were undertaken based on the premise that NO may be a critical mediator of renal haemodynamics. Our previous work has supported this proposal by demonstrating that inhibition of nitric oxide synthase (NOS) with L-NAME significantly reduced basal medullary blood perfusion [34]. Additionally, several prior studies alos demonstrated that NO plays a significant role in regulating renal vasculature tone in response to RNS [12,40].

In this study, inhibitor-NAME was administered locally into the cortico-medullary border to mitigate against any peripheral effects of NOS inhibition. When RNS was initiated in the presence of L-NAME, there was a modest increase in the reduction of both cortical and medullary blood perfusion compared to controls, particularly at higher frequencies. Although this change did not achieve statistical significance, it supports the hypothesis that NO may play a protective role in the renal medulla by preventing a substantial reduction in blood perfusion during RNS. If we had used an L-NAME concentration similar to that used in previous studies [12,40], we might have observed a more pronounced decrease in perfusion in response to RNS.

While NO contributes to the relative insensitivity of the medullary vasculature to RNS, it does not fully account for this phenomenon. Even with NOS inhibited, RNS still reduced CBP more than MBP, suggesting that as yet to be identified additional factors influence the observed responses. One possible factor is the innervation density of the juxtamedullary vasculature, which modulates blood flow into the medulla.

The findings from NOS blockade with L-NAME support the notion that NO partially mitigates RNS-mediated intra-renal vasoconstriction, thereby reducing the magnitude of the decrease in medullary blood perfusion. This effect is consistent with our previous results using normotensive rats.

## 4. Materials and Methods

All procedures were carried out in accordance with European Community Directive 86/609/EC and with the approval of the local Animal Experimentation Ethical Committee at University College Cork.

Male SHRSP rats weighing 250–300 g (approximately 12 weeks old) were sourced from Harlan (Bicester, UK), now known as Inotiv; the animal authorization number from Inotiv UK was PCD: 40/9001. The rats were housed in the Biological Services Unit at University College Cork for at least one week before experiments began. Animals had access to chow (Inotiv-Teklad, Leicestershire, UK) and water until 12 h before surgery. Anesthesia was induced with a 1 mL bolus of chloralose/urethane at 16.5/250 mg/mL and maintained intravenously with supplemental doses of 0.05 mL every 30 min.

The trachea was intubated using a short segment (3–4 cm) of polypropylene tubing (PP240, with an internal diameter of 1.67 mm and an external diameter of 2.42 mm, Portex Ltd., Harlow, Essex, UK). The tubing was secured to the trachea with thread and cut to terminate at the level of the nose, ensuring the animal’s normal dead space during breathing remained unchanged. The cannula aided respiration by maintaining a clear airway and allowing the removal of accumulated secretions when needed. Animals were permitted to breathe spontaneously in ambient air. The right femoral vein was cannulated for saline infusion at 3 mL/h and supplementary doses of anesthetic. A cannula was also placed in the right femoral artery to monitor blood pressure and heart rate continuously.

The left kidney was exposed, and an interstitial catheter was inserted approximately 5 mm into the lower pole of the kidney to enable the localized administration of either a vehicle or drugs into the renal cortical-medullary border (CMB) at a rate of 1 mL/h. This catheter was secured to the kidney surface with cyanoacrylate superglue (Bostik, Puteaux, France) and connected to a 2.5 mL Hamilton glass syringe (Model 100, KD Scientific, Holliston, MA, USA), set to deliver vehicle or drugs at a rate of 16.7 µL/min (1 mL/h).

Two optical fiber microprobes (MT B500-0 L120, 0.5 mm diameter, Perimed CE 0413, Stockholm, Sweden) were inserted into the kidney to depths of 1.5 mm to measure cortical blood perfusion (CBP), and 5.0 mm to measure medullary blood perfusion (MBP). The flow probes were connected to a laser-Doppler flowmeter (Periflex 4001 Master, Perimed, Stockholm, Sweden) and calibrated using a PF 1000 calibration solution (Perimed). After surgery, the animals were stabilized for 60 to 120 min before experimentation began.

After stabilization, bipolar stainless-steel electrodes were affixed to the fascia covering the renal artery and vein, where the renal sympathetic nerves are primarily situated. These electrodes were then connected to a stimulus isolation unit (Grass Medical Instrument, Model SIU 478 A, Quincy, MA, USA) and subsequently linked to an S8 Stimulator (Grass Medical Instruments, Quincy, MA, USA) which was set to generate square wave pulses with a duration of 2 ms and an amplitude of 15 V. Each stimulation period lasted for 1 min, with the renal nerves stimulated at frequencies of 0.5, 1, 2, 4, 6, or 8 Hz.

After the experiment, animals were euthanized with an anesthetic overdose, and the kidney was sectioned to confirm flow probe locations.

### 4.1. Drug Administration

#### 4.1.1. Control, Tempol, DETC and L-NAME Groups (*n* = 10 for Each Group)

The superoxide dismutase (SOD) mimetic 4-hydroxy-2,2,6,6-tetramethylpiperidin-1-oxyl (tempol; 30 μmol/kg/min), the SOD inhibitor Diethyldithio-carbamic acid (DETC; 2 mg/kg/min), and the non-selective NOS inhibitor NG-nitro-L-arginine-methyl ester (L-NAME; 10 μg/kg/min) were each dissolved in normal saline (0.9% NaCl) and infused into the cortico-medullary border at 1 mL/h. Normal saline was used as the vehicle for all experiments.

#### 4.1.2. Tempol Plus Catalase Group (*n* = 10)

Catalase, an enzyme that degrades H_2_O_2_ [37,38,41], was administered at 200 IU/kg/min 30 min before co-administration of tempol (30 μmol/kg/min) and catalase at 1 mL/h.

### 4.2. Experimental Protocol

Vehicle, tempol, DETC, and L-NAME were all administered using the same experimental protocol as follows:

The whole experimental protocol was divided into two parts:

Part 1: Baseline measurements of cortical and medullary blood perfusion, mean arterial pressure and heart rate were recorded and averaged over 2 min without renal nerve stimulation or drug intervention. After this assessment, renal nerve stimulation at 0.5 Hz was applied for 1 min, followed by a recovery period of 2 to 5 min. This process was repeated for stimulation at 1, 2, 4, 6, and 8 Hz, maintaining a 2 min baseline, stimulation, and recovery period before proceeding to the next frequency.

Part 2: This procedure phase mirrored Part 1 but occurred during drug or vehicle infusion at the cortico-medullary border.

The vehicle or drug was infused for 90 min. Following the infusion, baseline cortical and medullary blood perfusion, blood pressure, and heart rate recordings were obtained and averaged over 2 min. The nerve stimulation protocol was subsequently repeated.

The protocol for the combination infusion of tempol and catalase differed slightly. Catalase alone was infused for 30 min, followed by a co-infusion of tempol with catalase for 90 min. Following this, baseline recordings were retaken, and the previously described stimulation protocol for the other drugs was initiated.

### 4.3. Statistical Analysis

Data are presented as means ± standard error of the mean (SEM), with SEM serving as a measure of data dispersion. The significance of the changes was evaluated using a repeated-measures analysis of variance (ANOVA), followed by Bonferroni post hoc tests to compare replicate means. A classical one-way ANOVA was utilized to compare different groups, followed by Tukey’s test. An unpaired Student’s *t*-test was employed explicitly for comparisons between two distinct points in this study. A significance level of *p* < 0.05 was considered to be significant.

## 5. Conclusions

The results of this study demonstrate that manipulating the level of oxidative stress intra-renally significantly affects RNS-induced changes in cortical and medullary blood perfusion in SHRSPs.

Infusion of tempol into the cortico-medullary border did not significantly modify RNS-mediated reductions in cortical and medullary blood perfusion. Notably, the combination of tempol and catalase further attenuated the effects of RNS on both cortical and medullary blood perfusion, suggesting that H_2_O_2_-mediated oxidative stress plays a crucial role in mediating the impact of RNS on these parameters.

In contrast, renal infusion of DETC significantly attenuated the RNS-induced reductions in CBP and MBP, albeit to a lesser extent than the tempol plus catalase combination, highlighting the role of SOD inhibition in RNS-mediated hemodynamic responses.

Inhibiting NO production with L-NAME did not significantly alter the cortical and medullary blood perfusion response to RNS, which indicates that NO does not contribute significantly to protecting cortical and medullary blood perfusion from the consequences of RNS. However, further studies using greater L-NAME concentrations are required to confirm this observation.

This study on hypertensive rats demonstrated results similar to those previously reported in normotensive rats, indicating that RNS affects both strains similarly.

Overall, these findings support the notion that renal infusion of agents such as tempol, catalase, and DETC can modulate the hemodynamic effects of RNS, potentially through mechanisms involving oxidative stress. These observations could provide valuable insights for the generation of novel therapeutic strategies.

## Figures and Tables

**Table 1:** Shows the baseline values for blood pressure (BP), heart rate (HR), cortical blood perfusion (CBP) and medullary blood perfusion (MBP) in the vehicle, tempol, tempol + catalase, DETC and L-NAME groups before renal nerve stimulation at each of the frequencies listed.

Stimulation	Parameter	Vehicle	Tempol	Tempol Plus Catalase	DETC	L-NAME
**0.5 Hz**	BP (mmHg)	120 ± 3	129 ± 6	120 ± 6	130 ± 16	137 ± 4
	HR (B/min)	210 ± 17	205 ± 11	189 ± 18	187 ± 12	185 ± 20
	CBP (PU)	111 ± 15	112 ± 11	108 ± 22	105 ± 20	111 ± 19
	MBP (PU)	63 ± 14	59 ± 8	52 ± 6	54 ± 5	56 ± 8
**1.0 Hz**	BP (mmHg)	121 ± 3	129 ± 6	120 ± 7	129 ± 16	140 ± 5
	HR (B/min)	208 ± 17	203 ± 11	190 ± 18	187 ± 12	203 ± 16
	CBP (PU)	108 ± 13	111 ± 12	114 ± 23	106 ± 20	110 ± 19
	MBP (PU)	60 ± 12	57 ± 8	53 ± 6	55 ± 5	56 ± 8
**2.0 Hz**	BP (mmHg)	102 ± 3	129 ± 5	121 ± 7	129 ± 16	138 ± 5
	HR (B/min)	200 ± 17	199 ± 11	187 ± 17	187 ± 12	189 ± 22
	CBP (PU)	109 ± 14	110 ± 12	115 ± 23	105 ± 21	109 ± 19
	MBP (PU)	60 ± 14	59 ± 8	52 ± 6	54 ± 5	57 ± 8
**4.0 Hz**	BP (mmHg)	122 ± 2	130 ± 6	122 ± 8	127 ± 16	140 ± 5
	HR (B/min)	203 ± 17	201 ± 11	187 ± 18	187 ± 12	193 ± 19
	CBP (PU)	107 ± 13	109 ± 12	112 ± 23	101 ± 19	107 ± 18
	MBP (PU)	61 ± 14	50 ± 8	51 ± 5	55 ± 6	54 ± 8
**6.0 Hz**	BP (mmHg)	122 ± 3	132 ± 6	124 ± 8	127 ± 17	140 ± 5
	HR (B/min)	209 ± 16	202 ± 11	188 ± 18	168 ± 24	201 ± 17
	CBP (PU)	107 ± 14	109 ± 12	108 ± 22	98 ± 20	103 ± 20
	MBP (PU)	64 ± 15	55 ± 9	52 ± 5	53 ± 6	50 ± 6
**8.0 Hz**	BP (mmHg)	124 ± 3	132 ± 5	126 ± 8	125 ± 18	140 ± 5
	HR (B/min)	211 ± 17	200 ± 11	190 ± 19	166 ± 26	198 ± 17
	CBP (PU)	109 ± 14	107 ± 11	105 ± 20	95 ± 21	99 ± 21
	MBP (PU)	60 ± 17	57 ± 8	51 ± 6	53 ± 7	54 ± 7

## Data Availability

The original contributions presented in this study are included in the article. Further inquiries can be directed to the corresponding author.
